# The Principles of Surgical Treatment of Infections of the Peritoneum
1Opening Remarks at a Discussion at a Meeting of the Bristol Medico-Chirurgical Society, November 8th, 1922.


**Published:** 1923-01

**Authors:** Forbes Fraser

**Affiliations:** Surgeon, Royal United Hospital, Bath


### THE PRINCIPLES OF SURGICAL TREATMENT OF INFECTIONS OF THE PERITONEUM.[1]

BY

FORBES FRASER, C.B.E., F.R.C.S.,

*Surgeon, Royal United Hospital, Bath.*

TWENTY or thirty years ago, when abdominal operations of all kinds were of far less frequent occurrence than at the present time, peritoneal infection was looked on with grave anxiety by the surgeon, and the most strenuous efforts were made to limit by surgical measures the spread of inflammation, which it was felt Nature herself could do little to control.

For example, a case of acute appendicitis with peritonitis diffusing from the right iliac fossa, after removal of the appendix, was treated by thoroughly washing the inflamed peritoneum either with dilute antiseptics or with saline solution. Many surgeons went further and irrigated the

[1] *Opening Remarks at a Discussion at a Meeting of the Bristol Medico-Chirurgical Society, November 8th,* 1922.

whole peritoneal cavity with saline, even eviscerating the coils of intestine in order to cleanse them more effectively. Efforts were then made to allow of the escape of serous or purulent effusion by means of tubes inserted, not only in the neighbourhood of the appendix, but in the pelvis, in the loins and any other situations where the surgeon's experience led him to fear the accumulation of septic material.

As soon as possible after the patient recovered from the anæsthetic, and in fear of the onset of paralytic distension of the intestines, which might lead to fatal obstruction, calomel was administered, either in a few large doses or in frequent small ones. This was followed up by saline purgatives and by copious and repeated enemata.

To administer morphia in such a case was looked on as little less than a crime, and, indeed, the minds of surgeons were so imbued with the dangers of morphia in all abdominal operations that this drug was almost universally banned in abdominal surgery.

The practice of those days in the treatment of peritonitis may be shortly described as :—

1. Removal, when possible, of the main source of sepsis.

2. Cleansing of the peritoneal cavity by washing.

3. Drainage of the peritoneal cavity by tubes or other mechanical means.

4. Administration of purgative medicines.

5. Stern abstinence from morphia.

This practice was evidently based on the beliefs :—

1. That the peritoneum is vulnerable to infection and has little power of resisting and controlling it.

2. That intestines whose peritoneal coat is inflamed tend to become paralysed and, unless stimulated by artificial means, to pass into a condition of acute obstruction.

In other words, the treatment of five-and-twenty years ago was based on *profound distrust of the peritoneum.*

Doubts of the truth of the principles of that time and of the practice resulting therefrom began to assail my mind many years ago, and little by little those doubts have strengthened into a settled conviction, shared by many surgeons, that with the one exception of removal, when possible, of the main source of sepsis, treatment of peritonitis as carried out at that time was based on faulty reasoning and should be entirely altered.

My views have changed so completely that I am prepared to maintain the following propositions :—

1. That the peritoneum should never, or hardly ever, be washed.

2. That it should only seldom be drained.

3. That in peritonitis purgatives should never be administered.

4. That in peritonitis morphia should always be given.

It is therefore necessary to examine in detail the questions of *Washing, Draining* and *Purging.*

*Lavage of the Peritoneum.*

To inquire into the value of peritoneal lavage, it is necessary briefly to recall the nature of the protective mechanism of the peritoneum.

The latter is a large and complex space lined over its whole surface by a layer of endothelium whose continuity is interrupted only at the fimbriated orifices of the Fallopian tubes.

The importance of this endothelial lining has been aptly compared by Dudgeon and Sargent to that of the skin of the hand. While the skin is intact the hand may be plunged with comparative impunity into infective solutions of extreme virulence, but in the presence of the smallest abrasion grave infection is likely to ensue. Similarly, so long as the endothelial cells retain their vitality and continuity the peritoneum can deal with and control infection, but when a breach is produced by mechanical, chemical or bacterial agency a direct route is at once opened for the passage of the infecting agent into the blood-stream through the subendothelial vessels.

When pathogenic bacteria gain access to the peritoneal cavity two effects are produced :—

1. There is a rapid and copious pouring out of serous effusion containing phagocytic cells. With extraordinary rapidity the latter ingest invading bacteria and appear with them in the subendothelial lymph spaces on the surface of the diaphragm. The object of this protective effusion, therefore, is first to destroy the infecting agent, and then to carry it away from the peritoneal cavity.

2. The formation of a fine layer of fibrin over the intestinal surfaces. This layer is familiar to surgeons at operations in the form of plaques and masses of plastic lymph, but according to the researches of Dudgeon and Sargent it is everywhere present, and can be demonstrated by the microscope when it is too fine to be visible to the naked eye. The objects of this fibrinous layer are to protect the endothelium from the action of toxins, to enable the intestinal surfaces to adhere together in order mechanically to limit the spread of infection, and to provide fine strands or ladders along which phagocytes may travel on their way to the scene of action.

This, then, is the mechanism set to work by Nature to combat infections introduced into the peritoneal cavity.

When the latter is irrigated with saline solution the protective effusion, with its army of phagocytes, is washed away and the peritoneum is left bare of defence.

Were it possible completely to wash away the infection also this might be a justifiable proceeding, but it is altogether beyond the power of the most prolonged and thorough irrigation to cleanse and render sterile a cavity so large and complex and so full of nooks and crannies as that of the peritoneum.

In the second place, the peritoneum cannot be efficiently washed without introducing the irrigating tube, and probably also the hand, into every part of the abdomen ; and in this way not only will the endothelium inevitably be damaged, but the protective layer of fibrin will be disturbed and torn away, carrying further patches of endothelium with it and laying bare the vulnerable subendothelial tissue.

It is almost unnecessary to mention the added risk of carrying infection to areas hitherto unaffected.

It appears, therefore, that the more efficiently lavage is carried out the greater must be the harm that is done.

The undesirability of lavage as a routine measure in the treatment of ordinary cases of peritonitis is now recognised almost universally, and few surgeons at the present day would wash out the abdomen in generalising peritonitis from appendicitis, gangrenous gall-bladder, or perforation of a malignant growth of the colon. Nevertheless, in the treatment of perforated gastric and duodenal ulcers lavage is still not uncommonly practised, and it cannot be denied that in these cases good results are obtained under this method of treatment.

When we consider the disastrous results which attended lavage when it was extensively used in the more virulent infections, and the vastly improved mortality since its practice was discontinued, for these reasons, as well as on *a priori* pathological grounds, it must be concluded that in perforated gastric and duodenal ulcers lavage is unnecessary, that patients recover *in spite of* lavage and not by its aid, and that in these comparatively mild infections, as well as in the more virulent type, this method of treatment is best dispensed with.

While the peritoneal effusion is in the first place a powerful instrument of protection which should not be interfered with, it is obvious that in severe infections a point may be reached when the phagocytes have all been destroyed, and when the effusion has lost its protective properties and has itself become septic.

Recently a plea has been brought forward[1] for the use of lavage in the worst type of cases, when the patient appears to be overwhelmed with toxæmia. Willis records cases of severe infection in which he believes recovery to have been due to the use of saline irrigation with the definite object of getting rid of a mass of toxic fluid.

Whether equally good results might have been obtained by the mere removal of the effusion by soaking it up with gauze is a question which may justifiably be asked, in view of the disastrous effects of lavage in a large series of cases of septic peritonitis in which it was used at St. Thomas's Hospital some twenty years ago.

*Drainage of the Peritoneal Cavity.*

While peritoneal lavage has to a great extent been discarded, the use of peritoneal drainage still commands the confidence of so many surgeons, that to venture to express doubts of the efficacy of the drainage-tube in the treatment of peritonitis may well lay one open to the charge of dangerous surgical heresy. Nevertheless, it is necessary to inquire into the value or otherwise of this time-honoured practice.

To call to mind, once more, the reaction of serous membranes, including the peritoneum, when an irritant, infective, chemical or mechanical (such as a drainage-tube), is introduced into their interior—first an effusion, believed to be

[1] Willis, *Surg., Gynec. and Obst.*, 1921, xxxiii. 355.

protective, is poured out, and, second, around the site of irritation there takes place a rapid and profuse exudation of plastic lymph.

When a drainage-tube is inserted into the peritoneal cavity its first effect is to drain off some of the fluid—probably protective in its nature—from the neighbourhood of the tube. But this drainage, such as it is, can last only for a very short time, because within a few hours the tube will be completely encased in a layer of plastic lymph which completely shuts it off from the rest of the peritoneal cavity.

In order to verify this statement it is only necessary to open the abdomen of any patient who has died of septic peritonitis, the peritoneum having previously been drained. The tube will be found to be completely enclosed in plastic lymph, and to be draining, *not* the peritoneal cavity, but a localised abscess-cavity which surrounds it.

It therefore appears that an abscess-cavity has actually been brought about by the insertion of the drainage-tube, and that the surgeon, having carefully removed one source of infection, has been at pains to produce a second, only less dangerous because it communicates at one end with the surface of the body.

It may be argued that in the later stages of severe infection previously referred to, when the effusion has itself become a danger, the temporary escape allowed by the tube may be of great service to the patient, and may enable him to "turn the corner." This may be so, but in my opinion and experience the same result may equally be obtained by removal of the septic effusion at the time of operation by gently expressing it and soaking it up with gauze, so as to leave the peritoneum comparatively dry; by this means at least the formation of a secondary focus of infection, inevitable in the presence of a drainage-tube, may be avoided.

It must freely be acknowledged that excellent results are daily obtained in the treatment of peritonitis by peritoneal drainage. That drainage-tubes in the peritoneal cavity may do good in some way as yet unexplained is possible. They certainly do not act by draining the peritoneum.

Deliberate production of localised suppuration by means of a seton is an ancient practice, and the newer method of the fixation-abscess is said to be much in vogue at the present time amongst French physicians.

What may be the exact *modus operandi* of the seton and the fixation-abscess is scarcely a matter even of speculation for the mere surgeon. The action of the drainage-tube in the peritoneal cavity appears to be equally mysterious.

One of the most remarkable facts forced on operators who dealt extensively with the grossly infected wounds of the Great War was the extraordinary and unique power of serous membranes to withstand and control infection, provided the grosser sources of sepsis were removed—and subsequently the structures were given the opportunity of rest, and were not subjected to the irritation of foreign bodies, such as drainage-tubes.

The history of the knee-joint is well known—how in the early days, with touching faith in surgical tradition, we drained them with tubes and the limbs came off, till with increasing experience towards the end of the war it was universally looked on as a surgical crime to put a drainage-tube into a synovial cavity.

Experience of abdominal wounds was similar ; peritoneal cavities grossly soiled by fæces from punctured and lacerated bowels were found to do better if gently cleansed and closed without drainage.

In the treatment of abdominal wounds the tissue that required drainage was not the peritoneum, but the abdominal wall, and particularly the retro-peritoneal tissue.

Excluding early deaths from shock and hemorrhage, the major part of the mortality after operation resulted, not from peritonitis, but from spreading sepsis in the abdominal wall; and it was clearly shown that while the peritoneum itself has a wonderfully efficient protective mechanism, the retroperitoneal tissue and the intermuscular fascial planes are vulnerable structures which have little power of resistance. Therefore in dealing with septic peritonitis the abdominal wall should always be drained down to the peritoneum, but the peritoneal cavity itself should in general be closed without drainage.

Certain conditions, however, form exceptions to this rule, and in these the introduction of a drain into the peritoneum for a short time appears to be the correct procedure—

1. When there is free oozing at the site of operation which cannot be completely stopped. As a collection of blood-clot is obviously undesirable in the presence of infection a drain should be left in for 24–36 hours.

2. When it is expected that a viscus may discharge its contents at the site of operation; *e.g.* the ligature may cut through the softened cystic duct after removing a gangrenous gall-bladder, and bile may be discharged. A drain at the point of expected leakage is an obvious safeguard.

3. When there is present in the peritoneal cavity a localised abscess-cavity with organised walls which must discharge piecemeal over a period of time a drain must be left in. This is not strictly drainage of the peritoneum, but of an abscess-cavity which is really extra-peritoneal.

4. When on account of the patient's condition or for any other reason the primary focus of infection is not removed a drain must be inserted in its neighbourhood.

*Purgation.*

Of all the principles of surgery the greatest and the most universally applicable is that of *Rest to Inflamed Parts.* It is an interesting speculation to inquire why was it for so long the rule for surgeons to deny to the peritoneum that rest which it has been our aim to secure for all other structures when the seat of inflammation.

All aperients act as irritant poisons to the intestinal mucosa. They have been shown to increase the number and virulence of intestinal bacteria. By irritating the walls of the gut they tend to increase their penetration by germs, and by producing violent peristaltic contractions they disturb the protective layer of fibrin covering their peritoneal surfaces.

The dangers of giving aperients before operation have again and again been pointed out by Moynihan, who has convinced us that a mild septic focus may be transformed by their action into a fulminating lesion.

If dangerous before operation, why less so afterwards? It is true that the grosser source of sepsis is no longer present, but it is unlikely that the whole of the infection has been eliminated. Foci of sepsis in any acute case still remain, and in order that the protective forces of Nature may be given their fullest scope it is necessary that the peritoneum and intestines be kept at rest.

Two methods are at our disposal for giving rest to these organs :—

1. Abstinence from food.
2. Morphia.

The question of dietary after operation for peritonitis, as well as those of salines, the administration of glucose and alkalies, and of the Fowler position, is beyond the scope of these remarks, which will conclude with discussion of the use of morphia.

We have been told that morphia is dangerous, first because it increases the shock of operation, and also because it tends to produce paralytic ileus and intestinal obstruction.

With regard to shock, it is only necessary to say that the experience of years shows that morphia does not produce shock after operations of any kind when given in reasonably moderate doses, and further that Crile has proved that it is the one drug which has the power of diminishing shock.

With reference to paralytic ileus, one must again make a personal statement. For many years every patient after abdominal operation in my practice has had sufficient morphia to keep him in reasonable comfort when he needed it, and every case of peritonitis has had morphia as a routine for the first two or three days as part of his treatment, and whether he required it for pain or not.

While an ordinary incidence of flatulent distension occurs, easily relieved by flatus tube or by enema and pituitrin, the severe forms of paralytic ileus are practically unknown ; no case of ileus duplex has been seen.

In my earlier experience severe paralytic ileus was more common, before I habitually used morphia and while aperients were yet being given. Aperients, such as calomel, given while the bowels are inflamed actually tend to bring on the worst forms of intestinal distension, with the object of preventing which they were administered.

It will be noticed that pituitrin has been mentioned as being used for flatulence, and in clean operation cases it is of course a most valuable drug. But in cases of peritonitis I prefer to withhold even pituitrin for the first forty-eight hours if possible, and to keep the intestines absolutely at rest. For the same reason aperients are entirely forbidden till the first week has passed, though liquid paraffin is usually given twice a day after the first two or three days.

Earlier in these remarks it was pointed out that the treatment of peritonitis five-and-twenty years ago appeared to be based on *distrust* of the peritoneum and intestines, and of their capacity to take care of themselves after an operation for removal of the septic focus responsible for the onset of peritonitis.

The practice advocated now is based firstly on *profound faith* in the power of the peritoneum to resist and control infection, provided the main source of sepsis be removed and its mechanism be not interfered with by ungentle handling, by washing, by unnecessary drainage-tubes or other foreign bodies, or by the administration of irritant poisons in the form of aperients; secondly, on realisation of the truth and value of the great principle of *Rest to Inflamed Structures,* as set forth by John Hilton sixty years ago.

In conclusion, a few short extracts from his immortal lectures on *Rest and Pain*—perhaps the greatest classic of surgical therapeutics ever given to the world—require no apology for their quotation :—

" It would be well, I think, if the surgeon would fix upon his memory, as the first professional thought which should accompany him in the course of his daily occupation, this physiological truth—that Nature has a constant tendency to repair the injuries to which her structures have been subjected, whether those injuries be the result of fatigue or exhaustion, of inflammation or accident.

" Also that this recuperative power becomes at once most conspicuous when the disturbing cause has been removed . . ."

" That if the local disturbing cause, whatever it be, is removed, Nature has an immediate tendency to repair the injury which has been inflicted, because she is able to adopt her own remedy, Rest . . ."

"Nearly all our best considered operations are done for the purpose of making it possible to keep the structures at rest, or freeing Nature from the disturbing cause which was exhausting her powers, or making her repeated attempts at repair unavailing.

"So in all operations for hernia, the object is to give the intestine rest and freedom from pressure, both immediately after and for a long time subsequent to the operation. Most of us now act on this principle. We take care not only not to disturb the intestine by purgatives, but we give opium to arrest peristaltic action. We know the gut has been damaged in its function and structure and will not bear excitement; that it requires rest and quiet to enable it to repair itself."

---

